# A U.S. population‐based study of insurance disparities in cancer survival among adolescents and young adults

**DOI:** 10.1002/cam4.2230

**Published:** 2019-06-26

**Authors:** Meryl D. Colton, DeLayna Goulding, Alina Beltrami, Carrye Cost, Anna Franklin, Myles G. Cockburn, Adam L. Green

**Affiliations:** ^1^ University of Colorado School of Medicine Aurora Colorado; ^2^ Colorado School of Public Health Aurora Colorado; ^3^ University of Denver Denver Colorado; ^4^ Children's Hospital Colorado Aurora Colorado

**Keywords:** AYA, health disparities, insurance, SEER, stage

## Abstract

**Background:**

Adolescents and young adults (AYA), patients age 15‐39, may experience worse outcomes than pediatric and adult patients. The aim of this paper was to document survival disparities associated with insurance status across the AYA age continuum in the United States.

**Methods:**

We utilized the Surveillance, Epidemiologic, and End Results database to identify 66 556 AYA patients between 2007 and 2014 with 10 International Classification of Childhood Cancer diagnoses and calculated the Cox proportional hazard ratios of death for those with public or no insurance status compared to private insurance. The odds ratios of having a late stage of diagnosis by insurance status were also calculated.

**Results:**

Insurance status was a statistically significant predictor of death for lymphoid leukemia (age 15‐19, 30‐34, and 35‐39), acute myeloid leukemia (age 15‐19 and 25‐29), Hodgkin lymphoma (all ages), non‐Hodgkin lymphoma (age 20‐24, 25‐29, 30‐34, and 35‐39), astrocytomas (age 30‐34), other gliomas (age 25‐29, 30‐34, and 35‐39), hepatic carcinomas (age 25‐29), fibrosarcomas, peripheral nerve and other fibrous tumors (age 30‐34), malignant gonadal germ cell tumors (age 20‐24, 25‐29, 30‐34, and 35‐39), and other and unspecified carcinomas (age 20‐24, 25‐29, 30‐34, and 35‐39), independent of stage at diagnosis. This hazard increased with age for most cancer types. Insurance status strongly predicted the odds of a metastatic cancer diagnosis for lymphoma, fibrosarcomas (age 15‐19), germ cell tumors, and other carcinomas.

**Conclusions:**

AYA in the US experience disparities in cancer survival based on insurance status, independent of late stage of presentation. Patients age 26‐39 may be especially vulnerable to health outcomes associated with poor socioeconomic status, treatment disparities, and poor access to care.

## 1 INTRODUCTION

There are nearly 70 000 patients between the age of 15 and 39 diagnosed with cancer every year in the US, and cancer is the number one cause of disease‐related death among these adolescents and young adult patients (AYA).^1^ AYA cancer patients are a unique population who often experience worse outcomes than older or younger patients,^2^, ^3^ although not always.^4^ There are several potential reasons for these differences in survival, including differences in biology,^5^ poor access to regular health care services,^6^ inadequate inclusion in clinical trials,^7^, ^8^ and modest financial resources.^9^, ^10^

AYA in the US represent a diverse population with respect to social and financial resources.^11^ AYA populations experience significant transitions with education, employment, and family or partner relationships. Therefore, this population may be more susceptible to poor health outcomes associated with socioeconomic status (SES). SES is a predictor of failure to complete recommended therapy,^12^, ^13^ and AYA patients with greater financial stress may forgo medical treatments.^10^ The impact of socioeconomics on cancer mortality in AYA patients has not been well quantified, especially among rare cancers.

Insurance status, a key health‐related socioeconomic measure, also plays an important role in survival.^14^ Several cancers including germ cell tumors,^15^ thyroid cancer,^16^ lymphoma,^17^ and other solid tumors^18^, ^19^, ^20^ have worse outcomes for those with no insurance or public insurance compared to private insurance. The Patient Protection and Affordable Care Act (ACA)'s expansion of Medicaid and coverage to dependents under 26 years provided a recent means to protect this especially vulnerable age group.^19^, ^21^ Evidence suggests this disparity can, in part, be explained by a larger proportion of local stage diagnoses among those with private insurance in cancers that afford favorable prognosis when diagnosed at an early stage.^18^, ^22^, ^23^ However, it is not clear whether these patterns persist across cancer subtypes.

The aim of this analysis was to assess the role of insurance status on cancer survival among AYA with a broad group of cancer types common to the entire age range, including those that have improved outcomes when diagnosed at an early stage (eg, solid carcinomas) and those which early diagnosis does not improve survival (eg, gliomas). Furthermore, we aimed to test whether the potential relationship between insurance status and survival changes across the AYA age spectrum. Last, we explored reasons for the observed disparities in survival including stage of presentation.

## 2 METHODS

### 2.1 Patient population

We utilized the Surveillance, Epidemiologic, and End Results (SEER) 18 database, which covers approximately 28 percent of the United States population and is designed to represent both its geographical and social diversity. The SEER registries began collecting information on insurance in 2007. There were 1 19 612 patients between the age of 15 and 39 registered between 2007 and 2014 included in this analysis.

As the goal of this analysis was to provide a comprehensive analysis of insurance on cancer survival, we devised a systematic way to choose which cancer types to include. In short, we included each International Classification of Childhood Cancer (ICCC) subcategory for which there were at least five events in each insurance status by age bin, and there was complete information on stage at diagnosis. The ICCC categories were chosen to improve the chances of identifying more cancer subcategories with enough deaths across the AYA age spectrum, specifically the 15‐19 age group in which there are fewer deaths from solid tumors and other common AYA cancers. This included 10 ICCC subcategories: I(a) lymphoid leukemia, I(b) acute myeloid leukemia, II(a) Hodgkin lymphoma, II(b) Non‐Hodgkin lymphoma (except Burkitt lymphoma), III(b) astrocytomas, III(d) other gliomas, VII(b) hepatic carcinomas, IX(c) fibrosarcomas, peripheral nerve and other fibrous tumors, X(c) malignant gonadal germ cell tumors, and XI(f) other and unspecified carcinomas.

### 2.2 Key variables

Models were stratified by 5‐year age categories (ages 15‐19, 20‐24, 25‐29, 30‐34, and 35‐39). AYA were grouped into two insurance categories: private insurance and other, combining Medicaid and no insurance. Entries labeled “Insured/No specifics” were considered private insurance. Patients with Medicaid and no insurance were combined to improve the power of the analysis, since many patients are enrolled in Medicaid retrospectively after a cancer diagnosis, and other groups have utilized this grouping in analyses.^18^

Stage information was modified to a binary variable, local or metastatic. For Hodgkin and non‐Hodgkin lymphoma, local disease was considered stages I‐II, and metastatic was considered stages III‐IV based on AJCC Lymphoma staging. For brain tumors, local stage was considered “localized” and metastatic combined “regional” and “distant” using the Summary stage 2000 (1998+) variable, since both represent tumor that has spread elsewhere in the CNS. For the other disease categories (excluding the leukemias), local was considered “localized” and “regional,” and metastatic included only “distant” using the SEER historic stage A variable. Stage information was not included for models involving leukemia, which are metastatic by definition.

Data regarding race and ethnicity available through SEER include the race recode variable (White, Black, American Indian/Alaskan Native, Asian Pacific Islander) and Spanish‐Hispanic‐Latino ethnicity (Yes/No), which utilizes the NAACCR Hispanic Identification Algorithm (NHIA) to identify patients with Hispanic ancestry. We combined race and ethnicity for White non‐Hispanic, Black non‐Hispanic, and White Hispanic AYA. Patients not registered under these categories were combined into an “Other” category.

### 2.3 Statistical analysis

We calculated summary statistics by age categories. Univariate associations were performed using Fisher's exact tests to choose a limited number of variables for multivariable hazard ratio (HR) models. Variables with consistent associations (*P* < 0.05) were considered potential confounders and included in the multivariable analysis.

We calculated crude Cox proportional HR and 95% confidence intervals (95% CI) of death for those with Medicaid or no insurance compared to private insurance, stratified by age and ICCC category. We then compared crude HR and HR adjusted for race/ethnicity and stage at diagnosis to explore the influence of these factors. We also examined the HR of stage at diagnosis to further understand how stage at diagnosis mediates the effect of insurance status on death.

We calculated odds ratios (OR) of having metastatic disease at diagnosis by insurance status to comment further on this relationship.

All analyses were performed using SAS software version 9.4 (Cary, NC).

## 3 RESULTS

### 3.1 Patient population and characteristics

**Figure 1:**
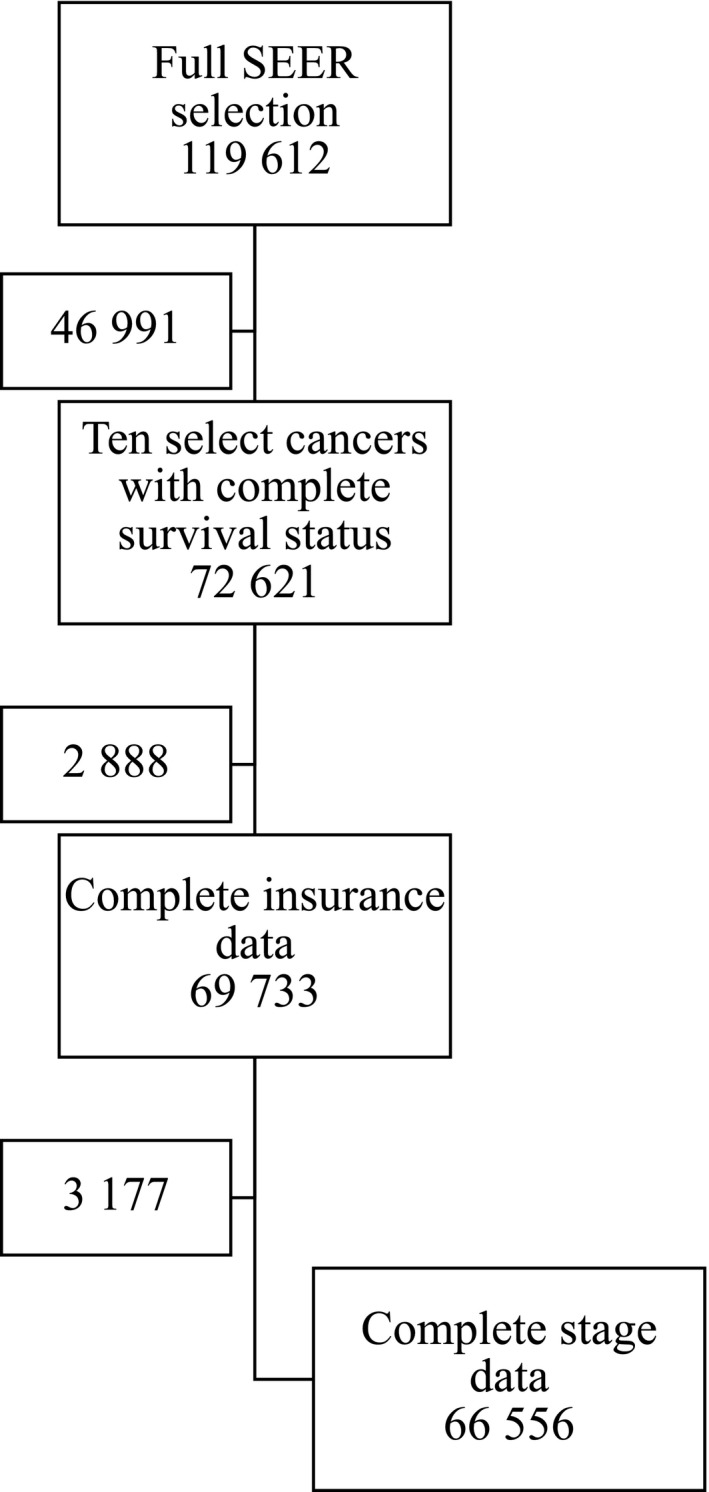
Description of patient participant selection

There were 1 19 612 AYA diagnosed with cancer between 2007 and 2014 in the SEER database. The selection for this analysis is presented in Figure 1. Complete information on insurance and stage were missing for 2888 and 3177 entries respectively, and these records were not included. Summary patient characteristics are detailed in Table 1. The ICCC category “Other and Unspecified Carcinomas” was the largest group. This group excludes adrenocortical carcinomas, thyroid carcinomas, nasopharyngeal carcinomas, malignant melanomas, and skin carcinomas but includes all other carcinomas including digestive tract, lung, urologic, and breast. Among the 15‐19‐year‐olds, Hodgkin lymphoma was the most prevalent cancer (24.9%). Malignant germ cell tumors were the most prevalent cancer among 20‐24‐year‐olds, and other and unspecified carcinomas were the most prevalent among 25‐29‐year‐olds, 30‐34‐year‐olds, and 35‐39‐year‐olds. Most patients had private insurance (72.6%) and were diagnosed with local stage disease (79.8%).

**Table 1:** Descriptive statistics by 5‐year age intervals

	Age (years)					
	15‐19	20‐24	25‐29	30‐34	35‐39	TOTAL
TOTAL						
N	4539	6992	10 848	16 747	27 430	66 556
Events n (%)	519 (11.4)	908 (13.0)	1457 (13.4)	2399 (14.3)	4389 (16.0)	9672 (14.5)
Insurance status						
Private insurance n (%)	3090 (68.1)	4692 (67.1)	7431 (68.5)	12 246 (73.1)	20 869 (76.1)	48 328 (72.6)
Public or no insurance n (%)	1449 (31.9)	2300 (32.9)	3417 (931.5)	4501 (26.9)	6561 (23.9)	18 228 (27.4)
Stage at presentation^a^						
Local n (%)	2569 (72.9)	4686 (74.8)	8015 (79.1)	12 915 (81.0)	21 494 (81.4)	49 679 (79.8)
Distant n (%)	953 (27.1)	1575 (25.2)	2120 (20.9)	3025 (19.0)	4924 (18.6)	12 597 (20.2)
Gender						
Female n (%)	1844 (40.6)	2702 (38.6)	5300 (48.9)	10 259 (61.3)	18 902 (68.9)	39 007 (58.6)
Male n (%)	2695 (59.4)	4290 (61.4)	5548 (51.1)	6488 (38.7)	8528 (31.1)	27 549 (41.4)
Race/Ethnicity						
NHW n (%)	2450 (53.9)	3877 (55.5)	6079 (56.0)	9168 (54.7)	15 360 (56.0)	36 934 (55.5)
HW n (%)	1248 (27.5)	1816 (25.9)	2521 (23.2)	3638 (21.72)	5275 (19.2)	14 498 (21.8)
NHB n (%)	425 (9.4)	652 (9.3)	1161 (10.7)	2032 (12.1)	3432 (12.5)	7702 (11.6)
Other n (%)	416 (9.17)	647 (9.3)	1087 (10.0)	1909 (11.4)	3363 (12.3)	7422 (11.15)
ICCC^b^ category						
I(a) Lymphoid leukemia n (%)	657 (14.5)	373 (5.3)	295 (2.7)	295 (1.8)	431 (1.6)	2051 (3.1)
I(b) Acute myeloid leukemia n (%)	360 (7.9)	358 (5.1)	418 (2.9)	512 (3.1)	581 (2.1)	2229 (3.4)
II(a) Hodgkin lymphomas n (%)	1128 (24.9)	1521 (21.8)	1398 (12.9)	1174 (7.0)	940 (3.4)	6161 (9.3)
II(b) Non‐Hodgkin lymphomas (except Burkitt lymphoma) n (%)	470 (10.4)	670 (9.6)	926 (8.5)	1240 (7.4)	1906 (7.0)	5212 (7.8)
III(b) Astrocytomas n (%)	421 (9.3)	396 (5.7)	485 (4.5)	553 (3.3)	646 (2.4)	2501 (3.8)
III(d) Other gliomas n (%)	133 (2.9)	185 (2.7)	298 (2.8)	395 (2.4)	364 (1.3)	1375 (2.1)
VII(b) Hepatic carcinomas n (%)	48 (1.1)	55 (0.8)	100 (0.9)	102 (0.6)	124 (0.5)	628 (0.9)
IX(b) Fibrosarcomas, peripheral nerve & other fibrous n (%)	54 (1.2)	71 (1.0)	81 (0.8)	142 (0.9)	283 (1.0)	432 (0.7)
X(c) Malignant gonadal germ cell tumors n (%)	878 (19.3)	2035 (29.1)	2614 (24.1)	2294 (3.5)	1835 (6.7)	9656 (14.5)
XI(f) Other and unspecified carcinomas n (%)	390 (8.6)	1328 (19.0)	4233 (39.0)	10 040 (60.0)	20 320 (74.0)	36 311 (54.6)

a Does not include leukemias.

b International Classification of Childhood Cancers (ICCC).

### 3.2 Insurance‐related risk of death

**Figure 2:**
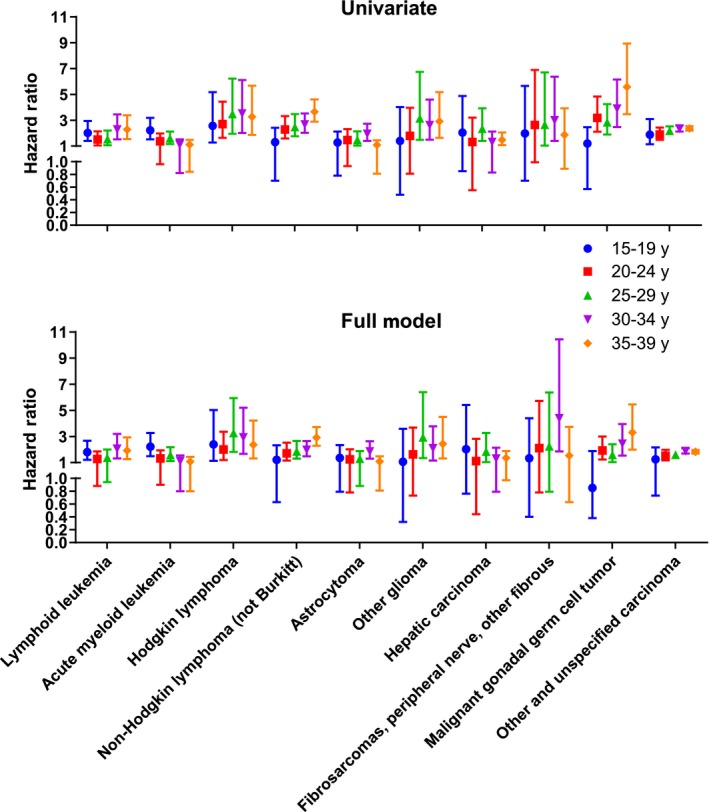
Crude (A) and adjusted (B) hazard ratios (with 95% confidence intervals) of death for those with public or no insurance compared to private insurance by 5‐year age intervals for 10 cancers

To determine the relationship between insurance status and death, we examined the HR of insurance status in crude and multivariable models by age group and ICCC category (Figure 2; HR and 95% CI available in Table S2). Univariate associations are summarized in Table S1 and illustrate consistent association between insurance status with race/ethnicity and stage. Consequently, multivariable models were adjusted for race/ethnicity and stage. There was an increased risk of death among those with public or no insurance compared to private insurance for most cancer types and age groups. This risk was mildly attenuated when adjusted for stage of presentation and race/ethnicity. A statistically significant increased risk of death persisted for lymphoid leukemia (ages 15‐19, 30‐34, and 35‐39), acute myeloid leukemia (ages 15‐19 and 25‐29), Hodgkin lymphoma (all ages), non‐Hodgkin lymphoma (ages 20‐24, 25‐29, 30‐34, and 35‐39), astrocytomas (age 30‐34), other gliomas (age 25‐29, 30‐34, and 35‐39), hepatic carcinoma (age 25‐29), fibrosarcoma, peripheral nerve, and other fibrous tumors (age 30‐34), malignant gonadal germ cell tumors (age 20‐24, 25‐29, 30‐34, and 35‐39) and other and unspecified carcinomas (age 20‐24, 25‐29, 30‐34, and 35‐39).

The largest hazards of death (with 95% CI) associated with public/no insurance in the multivariable models were among 25‐29‐year‐olds with Hodgkin lymphoma and other gliomas [3.27 (1.81, 5.94) and 2.93 (1.34, 6.39), respectively]. Among rarer cancers such as other gliomas, hepatic carcinomas, and fibrosarcomas/peripheral nerve/other fibrous tumors, this relationship was not statistically significant in each age category and there was also not a statistically significant relationship for patients aged 15‐19. The hazard of death among those with public or no insurance increased with age except for acute myeloid leukemia, astrocytomas, and hepatic carcinoma.

### 3.3 Metastatic stage of presentation‐related risk of death

We then investigated the HRs of metastatic vs local stage of presentation in the multivariable models to determine the effect of metastatic stage of presentation on death, independent of insurance status (Table 2). Univariate HRs and 95% CI are summarized in Table S3. There was a statistically significant increased risk of death with a metastatic stage of presentation for every category except astrocytomas (age 25‐29), other gliomas (age 15‐19, 20‐24, 25‐29, and 35‐39), and hepatic carcinomas (age 20‐24). The HRs of metastatic stage of presentation were largest among gonadal germ cell tumors and other and unspecified carcinomas. There was no pattern of increasing or decreasing risk of death associated with stage of presentation by age categories.

**Table 2:** Multivariable model HR and 95% CI of death for patients with metastatic stage of presentation compared to local stage of presentation by 5‐year age intervals for 10 cancers

	**15‐19**	**20‐24**	**25‐29**	**30‐34**	**35‐39**
II(a) Hodgkin lymphomas	**2.17 (1.06, 4.47)**	**2.24 (1.34, 3.74)**	**3.00 (1.64, 5.50)**	**2.93 (1.66, 5.20)**	**2.40 (1.32, 4.22)**
II(b) Non‐Hodgkin lymphomas (except Burkitt lymphoma)	**2.36 (1.26, 4.41)**	**2.99 (1.99, 4.49)**	**2.92 (2.00, 4.24)**	**2.42 (1.81, 3.23)**	**2.51 (1.94, 3.24)**
III(b) Astrocytomas	**5.80 (3.44, 9.80)**	**2.00 (1.08, 3.69)**	1.50 (0.91, 2.45)	**1.36 (0.88, 2.12)**	**1.66 (1.23, 2.25)**
III(d) Other gliomas	2.88 (0.57, 14.64)	2.36 (0.94, 5.94)	1.95 (0.77, 4.87)	**2.30 (1.22, 4.34)**	1.71 (0.90, 3.30)
VII(b) Hepatic carcinomas	**4.69 (1.85, 11.92)**	1.46 (0.54, 3.97)	**2.00 (1.18, 3.39)**	**2.71 (1.68, 4.38)**	**2.57 (1.84, 3.59)**
IX(b) Fibrosarcomas, peripheral nerve & other fibrous	**5.39 (1.43, 20.39)**	**7.46 (2.04, 27.24)**	**4.73 (1.73, 12.89)**	**8.1 (3.32, 20.05)**	**5.54 (2.95, 14.96)**
X(c) Malignant gonadal germ cell tumors	**4.25 (2.09, 8.63)**	**11.51 (7.25, 18.27)**	**13.07 (8.41, 20.29)**	**11.65 (7.23, 18.77)**	**19.09 (11.14, 32.71)**
XI(f) Other and unspecified carcinomas	**15.45 (8.99, 26.53)**	**12.94 (9.95, 16.84)**	**10.08 (8.63, 11.79)**	**9.64 (8.69, 10.69)**	**10.82 (10.06, 11.63)**

Notes

All HR adjusted for insurance status and race/ethnicity. Statistically significant results are highlighted in bold.

95% CI, 95% confidence intervals; HR, hazard ratios.

### 3.4 Odds of metastatic disease at diagnosis by insurance status

Next, we explored the odds of having metastatic disease at diagnosis for those with public or no insurance compared to private insurance (Table 3) to explore the role of metastatic disease presentation in explaining the effect of insurance status on death. We found increased odds of having a metastatic cancer diagnosis among those with public or no insurance with Hodgkin lymphoma, non‐Hodgkin lymphomas (age 20‐24, 25‐29, 30‐34, and 35‐39), fibrosarcomas/peripheral nerve/other fibrous tumors (age 15‐19), malignant gonadal germ cell tumors, and other and unspecified carcinomas. Notably, there were not significantly increased odds of having a metastatic cancer diagnosis among those with public or no insurance for those with astrocytomas, other gliomas, hepatic carcinomas, or fibrosarcomas/peripheral nerve/other fibrous tumors (age 20‐24, 25‐29, 30‐34, and 35‐39).

**Table 3:** Multivariable model OR and 95% CI of having a metastatic cancer diagnosis for those with public or no insurance compared to private insurance by 5‐year age intervals for 10 cancers

	**15‐19**	**20‐24**	**25‐29**	**30‐34**	**35‐39**
II(a) Hodgkin lymphomas	**1.63 (1.24, 2.13)**	**1.67 (1.33, 2.10)**	**1.59 (1.25, 2.02)**	**1.62 (1.23, 1.13)**	**2.15 (1.56, 2.98)**
II(b) Non‐Hodgkin lymphomas (except Burkitt lymphoma)	1.20 (0.91, 1.80)	**1.57 (1.15, 2.16)**	**1.48 (1.12, 1.97)**	**1.71 (1.33, 2.21)**	**1.63 (1.33, 2.01)**
III(b) Astrocytomas	1.02 (0.52, 2.02)	0.93 (0.49, 1.79)	1.23 (0.71, 2.18)	0.91 (0.53, 1.56)	0.94 (0.57, 1.53)
III(d) Other gliomas	3.75 (0.95, 14.85)	2.27 (0.93, 5.57)	1.50 (0.72, 3.15)	1.49 (0.77, 2.89)	1.62 (0.84, 3.13)
VII(b) Hepatic carcinomas	1.03 (0.30, 3.57)	2.76 (0.85, 9.01)	1.46 (0.63, 3.37)	1.71 (0.82, 3.57)	1.47 (0.83, 2.61)
IX(b) Fibrosarcomas, peripheral nerve & other fibrous	**5.63 (1.07, 29.73)**	1.25 (0.26, 6.07)	1.47 (0.34, 6.36)	0.78 (0.23, 2.64)	2.18 (0.75, 6.31)
X(c) Malignant gonadal germ cell tumors	**1.78 (1.27, 2.50)**	**2.04 (1.61, 2.58)**	**2.34 (1.86, 2.94)**	**2.29 (1.74, 2.99)**	**2.44 (1.78, 3.34)**
XI(f) Other and unspecified carcinomas	**1.87 (1.10, 3.17)**	**1.64 (1.25, 2.16)**	**1.78 (1.51, 2.09)**	**1.73 (1.55, 1.94)**	**1.96 (1.80, 2.12)**

Notes

All OR adjusted for insurance status and race/ethnicity. Statistically significant results are highlighted in bold.

95% CI, 95% confidence intervals; OR, odds ratios.

## 4 DISCUSSION

We used the SEER database to evaluate the hazard of death based on insurance status and stage at presentation among 66 556 AYA in a representative US population with 10 types of cancer by age category. Insurance status was a significant predictor of death for many age and cancer stratifications, independent of metastatic stage at diagnosis, and this hazard increased with age for most cancer types. Additionally, insurance status strongly predicted the odds of a metastatic cancer diagnosis for lymphoma, fibrosarcomas/peripheral nerve/other fibrous tumors (age 15‐19), germ cell tumors, and other carcinomas.

These findings add to the evidence showing an increased risk of death among those with public or no insurance compared to private insurance, independent of late stage of presentation. Rosenberg et al^18^ and Walker et al^19^ show similar increased risk of death for those with public or no insurance among common cancers that are known to have favorable prognosis when diagnosed at an early stage. This is the first study, to our knowledge that examines the effect of insurance status on survival across ICCC diagnoses that are common over the entire AYA age spectrum.

This study design allowed us to evaluate the effect of insurance status on survival based on age categories. These patterns help elucidate potential reasons for this disparity. For example, the risk of death for patients with public or no insurance increases with age among patients with acute lymphoblastic leukemia (ALL) but not acute myeloid leukemia (AML), potentially because ALL is more common in pediatric populations, and adult patients may benefit from specialized protocols derived for pediatric patients.^24^ This is consistent with the finding that the risk of death for those with public or no insurance does not increase with age for patients with astrocytomas, where most patients are diagnosed between ages 20 and 40, and the treatment for astrocytoma is more standardized throughout the age range.

The risk of death also increases with age for AYA with Hodgkin lymphoma. In recent years, the treatment regimens for Hodgkin lymphoma have changed between pediatric and adult patients, with adult patients receiving more radiation.^25^ Walker et al^19^ found that patients with Medicaid or no insurance were less likely to get radiotherapy, highlighting a potential reason for this increased risk of death.

Many older AYA (greater than age 26) with more aggressive cancers may benefit from clinical trials or treatment at a specialized cancer center. Alvarez et al^26^ found that between 1991 and 2014, AYAs in California with public or no insurance were less likely to receive care at a specialized cancer center compared to their privately insured counterparts. Furthermore, Lee et al^20^ found AYA with stage II‐III rectal cancer that were treated in a community center were less likely to receive recommended neoadjuvant chemotherapy and those with government insurance to be less likely to receive surgery, a major predictor of mortality. Increasing risk of death associated with insurance status may be explained by the fact that patients with public or no insurance have less access to these advanced, specialty resources, especially as they grow older.

In this study, we also saw that metastatic disease presentation is associated with decreased survival, independent of insurance status, for almost every cancer except other gliomas, highlighting the important role of diagnosing patients at the local disease stage.^27^ For lymphoma, fibrosarcoma (age 15‐19), germ cell tumors, and other carcinomas, there was a strong association between insurance status and the odds of metastatic disease presentation, suggesting that a portion of the crude effect of insurance status on survival could be mediated through the effect of metastatic presentation. This is consistent with Robbins et al,^22^ who found AYA with public or no insurance were more likely to have a metastatic cancer diagnosis. In this analysis, we show the association persists across fine age categories and among rare cancers, such as fibrosarcomas, in which local stage of diagnosis plays an important role in survival.

Since passage of the Patient Protection and Affordable Care Act in 2010, the number of 19‐25‐year‐olds with insurance coverage has increased as a result of the ACA's extended private insurance coverage to dependents under 26‐years‐old. This coverage is an important step to protect AYA; however, this analysis suggests that older AYA may be at greater risk for poor cancer survival outcomes if they do not have access to private insurance, and new policies are needed to protect this especially vulnerable age group.

There were several limitations of this study. The small sample size for some cancers (specifically other gliomas, hepatic cancer, and fibrosarcomas) limits our ability to definitely accept the null hypothesis. Furthermore, given this limited power, we did not choose to include potential confounders that have weak associations (such as marital status, rural and urban setting, etc) in the multivariable analysis. However, we do not expect these variables to play a significant role in the relationship between insurance status and survival.

Another limitation is the inability to distinguish between different private plans within private insurance. There are likely many factors within private plans that drive the given protection on survival. Further research should aim to understand these specific factors.

Last, this study was unable to differentiate between the effect of specific insurance provisions and the role of SES, since other individual‐level socioeconomic measures are not available through SEER. Consequently, associations with insurance status could be confounded with other socioeconomic factors that could modify survival such as poverty, education, and family support. This may be especially relevant for hepatocellular carcinoma, in which social factors play a large role in determining who is able to receive a liver transplant.^28^ A recent analysis of AYA in the California Cancer Registry demonstrated that insurance type modified neighborhood SES survival disparities^29^ suggesting that insurance plans are not just a marker of SES, but an important, independent factor in determining survival.

## 5 CONCLUSIONS

AYA with public or no insurance in a representative United States population are at increased risk of death, independent of stage of presentation, and this risk increases with age in cancers that are more common in younger patients. There are many possible reasons for this disparity, including access to advanced treatment plans, physician knowledge of treatment regimens, and socioeconomic factors. Patients age 26‐39 may experience particularly high risk when faced with a cancer diagnosis, and policies aimed at helping young adults should consider this vulnerable age group.
