# Supplementary material for: A U.S. population‐based study of insurance disparities in cancer survival among adolescents and young adults
Source: Cancer Med. 2019 Jun 26;8(10):4867–74. doi: 10.1002/cam4.2230 (PMC6712520; doi:10.1002/cam4.2230)
Supplement: Supplementary file 1 [file CAM4-8-4867-s001.docx]

Supplemental Data

| **Supplemental Table 1. Univariate relationships. P-value calculated using chi-squared tests** | | | | | | | | | | | | | | | | |
| --- | --- | --- | --- | --- | --- | --- | --- | --- | --- | --- | --- | --- | --- | --- | --- | --- |
|  | **Age 15-19** | | | | **Age 20-24** | | | | **Age 24-29** | | | | **Age 30-39** | | | |
|  | **PI** | **PU or NI** | **p-value** | **PI** | | **PU or NI** | **p-value** | **PI** | | **PU or NI** | **p-value** | **PI** | | **PU or NI** | **p-value** |  |
| **I(a) Lymphoid leukemia** |  |  |  |  | |  |  |  | |  |  |  | |  |  |  |
| **Female n (%)** | 123 (33.2) | 99 (34.6) | 0.695 | 65 (33.3) | | 63 (35.4) | 0.676 | 50 (33.3) | | 59 (40.7) | 0.191 | 201 (40.4) | | 88 (38.4) | 0.606 |  |
| **Race/Ethnicity n(%)** |  |  | **<.0001** |  | |  | **<.0001** |  | |  | **<.0001** |  | |  | **<.0001** |  |
| *Non-Hispanic White* | 198 (53.4) | 58 (20.3) |  | 96 (49.2) | | 49 (27.5) |  | 80 (53.3) | | 39 (26.9) |  | 292 (58.8) | | 55 (24.0) |  |  |
| *Hispanic White* | 121 (32.6) | 175 (6.2) |  | 64 (32.8) | | 108 (60.7) |  | 48 (32.0) | | 83 (57.2) |  | 116 (23.3) | | 121 (52.8) |  |  |
| Non-Hispanic Black | 19 (5.1) | 28 (9.8) |  | 8 (4.1) | | 11 (6.2) |  | 10 (6.7) | | 10 (6.9) |  | 35 (7.0) | | 28 (12.2) |  |  |
| Other | 33 (8.9) | 25 (8.7) |  | 27 (13.9) | | 10 (5.6) |  | 12 (8.) | | 13 (9.0) |  | 54 (10.9) | | 25 (10.9) |  |  |
| **I(b) Acute myeloid leukemia** |  |  |  |  | |  |  |  | |  |  |  | |  |  |  |
| **Female n(%)** | 114 (49.6) | 74 (56.9) | 0.179 | 103 (49.1) | | 82 (55.4) | 0.236 | 133 (50.9) | | 68 (43.3) | 0.129 | 392 (52.8) | | 163 (46.4) | **0.048** |  |
| **Race/Ethnicity n(%)** |  |  | **<.0001** |  | |  | **<.0001** |  | |  | **<.0001** |  | |  | **<0.001** |  |
| *Non-Hispanic White* | 151 (65.7) | 44 (33.9) |  | 132 (62.9) | | 43 (29.1) |  | 145 (55.6) | | 65 (41.4) |  | 423 (57.0) | | 127 (36.2) |  |  |
| *Hispanic White* | 36 (15.7) | 54 (41.5) |  | 38 (18.1) | | 64 (43.2) |  | 53 (20.3) | | 57 (36.3) |  | 136 (18.3) | | 133 (37.9) |  |  |
| Non-Hispanic Black | 18 (7.8) | 13 (10.0) |  | 15 (5.1) | | 28 (18.9) |  | 22 (8.4) | | 24 (15.3) |  | 69 (9.3) | | 56 (15.9) |  |  |
| Other | 25 (10.9) | 19 (14.6) |  | 25 (11.9) | | 13 (8.8) |  | 41 (15.7) | | 11 (7.0) |  | 114 (15.4) | | 35 (9.9) |  |  |
| **II(a) Hodgkin lymphomas** |  |  |  |  | |  |  |  | |  |  |  | |  |  |  |
| **Distant Stage at Diagnosis n(%)** | 277 (22.5) | 135 (45.0) | **<0.001** | 364 (33.0) | | 188 (45.0) | **<0.001** | 322 (31.9) | | 165 (42.6) | **<0.001** | 535 (32.5) | | 219 (46.9) | **<0.001** |  |
| **Female n (%)** | 419 (50.6) | 148 (49.3) | 0.706 | 552 (50.0) | | 208 (49.9) | 0.967 | 515 (50.9) | | 181 (46.8) | 0.163 | 753 (45.7) | | 193 (41.3) | 0.092 |  |
| **Race/Ethnicity n(%)** |  |  | **<0.001** |  | |  | **<0.001** |  | |  | **<0.001** |  | |  | **<0.001** |  |
| *Non-Hispanic White* | 577 (69.7) | 126 (42) |  | 731 (66.2) | | 200 (48.0) |  | 693 (68.6) | | 189 (48.8) |  | 1127 (68.4) | | 220 (47.1) |  |  |
| *Hispanic White* | 188 (14.3) | 94 (31.3) |  | 156 (14.1) | | 120 (28.8) |  | 116 (11.5) | | 84 (21.7) |  | 205 (12.5) | | 125 (26.8) |  |  |
| Non-Hispanic Black | 68 (8.2) | 50 (16.7) |  | 104 (9.4) | | 74 (17.8) |  | 107 (10.6) | | 90 (23.3) |  | 194 (11.8) | | 100 (21.4) |  |  |
| Other | 65 (7.9) | 30 (10.0) |  | 113 (10.2) | | 23 (5.5) |  | 95 (9.40) | | 24 (6.2) |  | 121 (7.4) | | 22 (4.71) |  |  |
| **II(b) Non-Hodgkin lymphomas (except Burkitt lymphoma)** |  |  |  |  | |  |  |  | |  |  |  | |  |  |  |
| **Distant Stage at Diagnosis n(%)** | 150 (44.6) | 66 (49.3) | 0.365 | 176 (41.4) | | 129 (52.7) | **0.005** | 274 (42.2) | | 144 (52.0) | **0.006** | 990 (42.3) | | 442 (54.9) | **<0.001** |  |
| **Female n (%)** | 125 (37.2) | 40 (29.9) | 0.132 | 169 (39.8) | | 83 (33.9) | 0.13 | 290 (44.7) | | 89 (32.1) | **<0.001** | 1022 (43.7) | | 280 (34.8) | **<0.001** |  |
| **Race/Ethnicity n(%)** |  |  | **<0.001** |  | |  | **<0.001** |  | |  | **<0.001** |  | |  | **<0.001** |  |
| *Non-Hispanic White* | 212 (63.1) | 48 (35.8) |  | 257 (60.5) | | 76 (31.0) |  | 395 (60.9) | | 99 (35.7) |  | 1425 (60.9) | | 288 (35.8) |  |  |
| *Hispanic White* | 51 (15.2) | 52 (38.8) |  | 67 (15.8) | | 91 (37.1) |  | 104 (16.0) | | 95 (34.3) |  | 373 (15.9) | | 263 (32.7) |  |  |
| Non-Hispanic Black | 45 (13.4) | 21 (15.7) |  | 52 (12.2) | | 53 (21.6) |  | 79 (12.2) | | 63 (22.7) |  | 279 (11.9) | | 196 (24.4) |  |  |
| Other | 28 (8.3) | 13 (9.7) |  | 49 (11.5) | | 25 (10.2) |  | 71 (10.9) | | 20 (7.2) |  | 264 (11.3) | | 58 (7.2) |  |  |
| **III(b) Astrocytomas** |  |  |  |  | |  |  |  | |  |  |  | |  |  |  |
| **Distant Stage at Diagnosis n(%)** | 32 (10.6) | 13 (10.8) | 0.952 | 33 (12.4) | | 15 (11.6) | 0.83 | 34 (10.9) | | 23 (13.2) | 0.453 | 131 (15.1) | | 46 (13.9) | 0.602 |  |
| **Female n (%)** | 144 (47.8) | 57 (47.5) | 0.95 | 132 (49.4) | | 52 (40.3) | 0.088 | 131 (42.1) | | 64 (36.8) | 0.25 | 341 (39.3) | | 130 (39.3) | 0.997 |  |
| **Race/Ethnicity n(%)** |  |  | **<0.001** |  | |  | **<0.001** |  | |  | **<0.001** |  | |  | **<0.001** |  |
| *Non-Hispanic White* | 212 (70.4) | 36 (30.0) |  | 202 (75.7) | | 62 (48.1) |  | 232 (74.6) | | 89 (41.2) |  | 615 (70.9) | | 157 (47.4) |  |  |
| *Hispanic White* | 31 (10.3) | 46 (38.3) |  | 30 (11.2) | | 34 (26.4) |  | 36 (11.6) | | 47 (27) |  | 120 (13.8) | | 85 (25.7) |  |  |
| Non-Hispanic Black | 21 (7.0) | 21 (17.5) |  | 14 (5.2) | | 21 (16.3) |  | 13 (4.2) | | 23 (13.2) |  | 40 (4.6) | | 57 (17.2) |  |  |
| Other | 37 (12.3) | 17 (14.2) |  | 21 (7.9) | | 12 (9.30 |  | 30 (9.7) | | 15 (8.6) |  | 93 (10.7) | | 32 (9.7) |  |  |
| **III(d) Other gliomas** |  |  |  |  | |  |  |  | |  |  |  | |  |  |  |
| **Distant Stage at Diagnosis n(%)** | 4 (4.1) | 5 (13.9) | **0.046** | 13 (9.7) | | 10 (19.6) | 0.068 | 21 (10.1) | | 13 (14.4) | 0.278 | 80 (13.3) | | 30 (19.0) | 0.071 |  |
| **Female n (%)** | 37 (38.1) | 18 (50.0) | 0.217 | 60 (44.8) | | 30 (58.8) | 0.088 | 93 (44.7) | | 42 (46.7) | 0.756 | 263 (43.8) | | 65 (41.1) | 0.554 |  |
| **Race/Ethnicity n(%)** |  |  | **<0.001** |  | |  | **0.027** |  | |  | **0.012** |  | |  | **<0.001** |  |
| *Non-Hispanic White* | 78 (80.4) | 12 (33.3) |  | 92 (68.7) | | 26 (50.9) |  | 160 (76.7) | | 54 (60.) |  | 428 (71.2) | | 77 (48.7) |  |  |
| *Hispanic White* | 8 (8.3) | 8 (22.2) |  | 18 (13.4) | | 16 (31.4) |  | 33 (15.9) | | 24 (26.7) |  | 68 (11.30 | | 9 (5.7) |  |  |
| Non-Hispanic Black | 4 (4.1) | 13 (36.1) |  | 9 (6.7) | | 5 (9.8) |  | 5 (2.4) | | 7 (7.8) |  | 33 (5.5) | | 17 (10.8) |  |  |
| Other | 7 (7.2) | 3 (8.3) |  | 15 (11.2) | | 4 (7.8) |  | 10 (4.8) | | 5 (5.6) |  | 68 (11.3) | | 9 (5.7) |  |  |
| **VII(b) Hepatic carcinomas** |  |  |  |  | |  |  |  | |  |  |  | |  |  |  |
| **Distant Stage at Diagnosis n(%)** | 9 (31.0) | 6 (31.6) | 0.968 | 8 (22.9) | | 9 (45.0) | 0.087 | 18 (30.5) | | 16 (39.0) | 0.377 | 73 (24.7) | | 44 (34.1) | **0.045** |  |
| **Female n (%)** | 12 (41.4) | 10 (52.6) | 0.444 | 16 (45.7) | | 6 (30.0) | 0.253 | 22 (37.3) | | 11 (26.8) | 0.274 | 117 (39.5) | | 32 (24.8) | **0.004** |  |
| **Race/Ethnicity n(%)** |  |  | 0.159 |  | |  | 0.086 |  | |  | **0.005** |  | |  | **0.015** |  |
| *Non-Hispanic White* | 16 (55.2) | 6 (31.6) |  | 23 (65.7) | | 6 (30.0) |  | 27 (45.8) | | 11 (26.8) |  | 108 (36.5) | | 36 (27.9) |  |  |
| *Hispanic White* | 4 (13.8) | 8 (42.1) |  | 6 (17.1) | | 7 (35.0) |  | 7 (11.9) | | 15 (36.6) |  | 38 (12.8) | | 31 (24.0) |  |  |
| Non-Hispanic Black | 5 (17.2 ) | 3 (15.8) |  | 3 (8.6) | | 3 (15.0) |  | 7 (11.9) | | 9 (22.0) |  | 38 (12.8) | | 31 (24.0) |  |  |
| Other | 4 (13.8) | 2 (10.5) |  | 3 (8.6) | | 4 (20.0) |  | 18 (30.5) | | 6 (14.6) |  | 105 (35.5) | | 38 (29.5) |  |  |
| **IX(b) Fibrosarcomas, peripheral nerve & other fibrous** |  |  |  |  | |  |  |  | |  |  |  | |  |  |  |
| **Distant Stage at Diagnosis n(%)** | 3 (7.3) | 4 (30.8) | **0.028** | 4 (9.1 ) | | 3 (11.1) | 0.782 | 7 (11.3) | | 3 (15.8) | 0.602 | 23 (13.9) | | 11 (18.0) | 0.445 |  |
| **Female n (%)** | 23 (56) | 5 (38.5) | 0.268 | 23 (52.3) | | 11 (40.7) | 0.345 | 32 (51.6) | | 11 (57.9) | 0.631 | 74 (44.9) | | 32 (52.5) | 0.309 |  |
| **Race/Ethnicity n(%)** |  |  | **0.031** |  | |  | **0.031** |  | |  | 0.628 |  | |  | **0.003** |  |
| *Non-Hispanic White* | 27 (65.9) | 3 (23.1) |  | 27 (65.9) | | 3 (23.1) |  | 23 (52.3) | | 11 (40.7) |  | 45 (72.6) | | 6 (31.6) |  |  |
| *Hispanic White* | 10 (24.4) | 5 (38.5) |  | 10 (24.4) | | 5 (38.5) |  | 9 (20.5) | | 9 (33.3) |  | 12 (19.4) | | 8 (42.1) |  |  |
| Non-Hispanic Black | 3 (7.3) | 4 (30.8) |  | 3 (7.3) | | 4 (30.8) |  | 8 (18.2) | | 4 (14.8) |  | 3 (4.8) | | 5 (26.2) |  |  |
| Other | 1 (2.4) | 1 (7.7) |  | 1 (2.4) | | 1 (7.7) |  | 4 (9.1) | | 3 (11.1) |  | 2 (3.2) | | 0 (0.0) |  |  |
| **X(c) Malignant gonadal germ cell tumors** |  |  |  |  | |  |  |  | |  |  |  | |  |  |  |
| **Stage at Diagnosis n(%)** | 99 (16.9) | 78 (26.6) | **<0.001** | 194 (13.9) | | 159 (24.8) | **<0.001** | 196 (10.5) | | 161 (21.5) | **<0.001** | 298 (9.2) | | 170 (19.2) | **<0.001** |  |
| **Female n (%)** | 109 (18.6) | 59 (20.1) | 0.593 | 113 (8.1) | | 55 (8.6) | 0.718 | 89 (4.8) | | 49 (6.6) | 0.066 | 116 (3.6) | | 40 (4.5) | 0.192 |  |
| **Race/Ethnicity n(%)** |  |  | **<0.001** |  | |  | **<0.001** |  | |  | **<0.001** |  | |  | **<0.001** |  |
| *Non-Hispanic White* | 361 (61.7) | 83 (28.3) |  | 920 (66.0) | | 230 (35.9) |  | 1240 (66.5) | | 363 (48.7) |  | 236 (72.8) | | 457 (51.7) |  |  |
| *Hispanic White* | 162 (27.7) | 176 (60.1) |  | 339 (24.3) | | 343 (53.5) |  | 429 (23.0) | | 315 (42.1) |  | 554 (17.1) | | 313 (35.4) |  |  |
| Non-Hispanic Black | 17 (2.9) | 16 (5.5) |  | 33 (2.4) | | 25 (3.9) |  | 35 (1.9) | | 34 (3.2) |  | 87 (2.7) | | 60 (6.8) |  |  |
| Other | 45 (7.7) | 18 (6.14) |  | 102 (7.3) | | 43 (6.7) |  | 162 (8.7) | | 45 (6.0) |  | 242 (7.5) | | 55 (6.2) |  |  |
| **XI(f) Other and unspecified carcinomas** |  |  |  |  | |  |  |  | |  |  |  | |  |  |  |
| **Distant Stage at Diagnosis (n(%)** | 42 (15.4) | 30 (25.4) | **0.02** | 155 (17.5) | | 115 (25.9) | **<0.001** | 408 (14.3) | | 315 (22.8) | **<0.001** | 3107 (13.7) | | 1750 (22.9) | **<0.001** |  |
| **Female n (%)** | 168 (61.8) | 60 (50.9) | **0.045** | 569 (64.4) | | 310 (69.8) | **0.048** | 2267 (79.4) | | 1104 (80.1) | 0.636 | 18764 (82.6) | | 6095 (79.7) | **<0.001** |  |
| **Race/Ethnicity n(%)** |  |  | **<0.001** |  | |  | **<0.001** |  | |  | **<0.001** |  | |  | **<0.001** |  |
| *Non-Hispanic White* | 164 (60.3) | 38 (32.2) |  | 543 (61.4) | | 155 (34.9) |  | 1654 (57.9) | | 492 (35.7) |  | 13288 (58.5) | | 2913 (38.1) |  |  |
| *Hispanic White* | 47 (17.3) | 42 (35.6) |  | 153 (17.3) | | 144 (32.4) |  | 503 (17.6) | | 452 (32.8) |  | 3570 (15.7) | | 2561 (33.5) |  |  |
| Non-Hispanic Black | 35 (12.9) | 21 (17.8) |  | 84 (9.5) | | 98 (22.1) |  | 327 (11.5) | | 98 (21.6) |  | 2687 (11.8) | | 1428 (18.7) |  |  |
| Other | 26 (9.6) | 17 (14.4) |  | 104 (11.8) | | 47 (106) |  | 370 (13.0) | | 137 (9.9) |  | 3169 (13.9) | | 744 (9.7) |  |  |

| **Supplemental Table 2. Univariate hazard ratios (HR) and 95% confidence intervals (95%CI) of death for those with public or no insurance compared to private insurance** | | | | |
| --- | --- | --- | --- | --- |
|  | **Age 15-19** | **Age 20-24** | **Age 25-29** | **Age 30-39** |
| **I(a) Lymphoid leukemia** | **2.01 (1.39, 2.93)** | **1.50 (1.05, 2.14)** | **1.53 (1.06, 2.20)** | **2.30 (1.74, 3.06)** |
| **I(b) Acute myeloid leukemia** | **2.21 (1.52, 3.19)** | 1.37 (0.96, 1.97) | **1.60 (1.16, 2.12)** | 1.10 (0.89, 1.35) |
| **II(a) Hodgkin lymphomas** | **2.56 (1.27, 5.18)** | **2.7 (1.64, 4.44)** | **3.48 (1.95, 6.22)** | **3.32 (2.25, 4.91)** |
| **II(b) Non-Hodgkin lymphomas (except Burkitt lymphoma)** | 1.30 (0.70, 2.42) | **2.29 (1.58, 3.32)** | **2.47 (1.76, 3.48)** | **3.21 (2.68, 3.83)** |
| **III(b) Astrocytomas** | 1.28 (0.78, 2.12) | 1.46 (0.93, 2.31) | **1.48 (1.03, 2.13)** | **1.35 (1.09, 1.67)** |
| **III(d) Other gliomas** | 1.40 (0.48, 4.02) | 1.79 (0.81, 3.96) | **3.15 (1.48, 6.75)** | **2.74 (1.83, 4.08)** |
| **VII(b) Hepatic carcinomas** | 2.04 (0.85, 4.88) | 1.32 (0.55, 3.20) | **2.34 (1.39, 3.93)** | **1.44 (1.10, 1.88)** |
| **IX(b) Fibrosarcomas, peripheral nerve & other fibrous** | 1.98 (0.70, 5.66) | 2.63 (0.99, 6.90) | **2.64 (1.04, 6.70)** | **2.38 (1.41, 4.03)** |
| **X(c) Malignant gonadal germ cell tumors** | 1.19 (0.57, 2.46) | **3.19 (2.11, 4.84)** | **2.84 (1.90, 4.25)** | **4.59 (3.30, 6.37)** |
| **XI(f) Other and unspecified carcinomas** | **1.88 (1.14, 3.09)** | **1.90 (1.48, 2.43)** | **2.17 (1.87, 2.53)** | **2.35 (2.22, 2.49)** |

| **Supplemental Table 3. Multiariable hazard ratios (HR) and 95% confidence intervals (95%CI) of death for those with public or no insurance compared to private insurance** | | | | |
| --- | --- | --- | --- | --- |
|  | **Age 15-19** | **Age 20-24** | **Age 25-29** | **Age 30-39** |
| **I(a) Lymphoid leukemia** | **1.80 (1.21 ,2.68)** | 1.28 (0.88, 1.85) | 1.37 (0.94, 2.00) | **1.97 (1.45, 2.67)** |
| **I(b) Acute myeloid leukemia** | **2.21 (1.49, 3.27)** | 1.32 (0.90, 1.94) | **1.57 (1.12, 2.18)** | 1.07 (0.86, 1.33) |
| **II(a) Hodgkin lymphomas** | **2.39 (1.13, 5.02)** | **2.00 (1.19, 3.36)** | **3.27 (1.81, 5.94)** | **2.65 (1.77, 3.98)** |
| **II(b) Non-Hodgkin lymphomas (except Burkitt lymphoma)** | 1.21 (0.63, 2.32) | **1.70 (1.15, 2.52)** | **1.86 (1.30, 2.66)** | **2.49 (2.07, 3.00)** |
| **III(b) Astrocytomas** | 1.36 (0.79, 2.34) | 1.25 (0.78, 2.02) | 1.29 (0.88, 1.88) | **1.32 (1.06, 1.66)** |
| **III(d) Other gliomas** | 1.06 (0.32, 3.59) | 1.63 (0.73, 3.68) | **2.93 (1.34, 6.39)** | **2.31 (1.51, 3.51)** |
| **VII(b) Hepatic carcinomas** | 2.03 (0.76, 5.42) | 1.12 (0.44, 2.82) | **1.84 (1.03, 3.27)** | 1.29 (0.98, 1.69) |
| **IX(b) Fibrosarcomas, peripheral nerve & other fibrous** | 1.33 (0.40, 4.39) | 2.11 (0.78, 5.73) | 2.24 (0.79, 6.37) | **2.41 (1.35, 4.33)** |
| **X(c) Malignant gonadal germ cell tumors** | 0.85 (0.38, 1.88) | **1.92 (1.23, 3.00)** | **1.58 (1.04, 2.40)** | **2.81 (1.99, 3.96)** |
| **XI(f) Other and unspecified carcinomas** | 1.25 (0.73, 2.16) | **1.52 (1.17, 1.98)** | **1.60 (1.37, 1.87)** | **1.84 (1.73, 1.96)** |
